# Dissemination of High-Risk Clones *Enterobacterales* among Bulgarian Fecal Carriage Isolates

**DOI:** 10.3390/microorganisms10112144

**Published:** 2022-10-29

**Authors:** Rumyana Markovska, Petya Stankova, Temenuga Stoeva, Marianna Murdjeva, Yulia Marteva-Proevska, Dobrinka Ivanova, Maryia Sredkova, Atanaska Petrova, Kalina Mihova, Lyudmila Boyanova

**Affiliations:** 1 Department of Medical Microbiology, Medical Faculty, Medical University of Sofia, 1432 Sofia, Bulgaria; 2 Department of Microbiology and Virology, University Multiprofile Hospital for Active Treatment (UMHAT) “St. Marina”—Varna, Medical University of Varna, 9002 Varna, Bulgaria; 3 Department of Microbiology and Immunology, University Multiprofile Hospital for Active Treatment (UMHAT) “St. George” Plovdiv, Medical University of Plovdiv, 4002 Plovdiv, Bulgaria; 4 Department of Medical Microbiology, Medical Faculty, University Multiprofile Hospital for Active Treatment (UMHAT) Alexandrovska, Medical University of Sofia, 1431 Sofia, Bulgaria; 5 Second Multiprofile Hospital for Active Treatment, 1202 Sofia, Bulgaria; 6 Department of Microbiology and Virology, University Multiprofile Hospital for Active Treatment (UMHAT) “Georgi Stranski”, Medical University of Pleven, 5800 Pleven, Bulgaria; 7 Molecular Medicine Center, Medical University of Sofia, 1432 Sofia, Bulgaria

**Keywords:** fecal carriage, ESBL, carbapenemases, NDM, KPC, Bulgaria

## Abstract

The gastrointestinal tract is an important reservoir of high-risk Enterobacteria clones and a driver of antimicrobial resistance in hospitals. In this study, patients from six hospitals in four major Bulgarian towns were included in this study. Overall, 205 cefotaxime-resistant isolates (35.3%) of *Enterobacterales* order were detected in fecal samples among 580 patients during the period of 2017–2019. ESBL/carbapenemase/plasmidic AmpC producer rates were 28.8%, 2.4%, and 1.2%, respectively. A wide variety of ESBLs: CTX-M-15 (41%), CTX-M-3 (24%), CTX-M-27 (11%), and CTX-M-14 (4%) was found. The carbapenemases identified in this study were New Delhi metalo-β-lactamase (NDM)-1 (5.4%) and *Klebsiella* carbapenemase (KPC)-2 (1.5%). Most NDM-1 isolates also produced CTX-M-15/-3 and CMY-4 β-lactamases. They belonged to ST11 *Klebsiella pneumoniae* clone. The epidemiology typing revealed three main high-risk *K. pneumoniae* clones (26%)—ST11, ST258, and ST15 and five main *Escherichia coli* clones—ST131 (41.7%), ST38, ST95, ST405, and ST69. Sixty-one percent of ST131 isolates were from the highly virulent epidemic clone O25b:H4-ST131. Phylotyping revealed that 69% of *E. coli* isolates belonged to the virulent B2 and D groups. Almost all (15/16) *Enterobacter* isolates were identified as *E. hormaechei* and the most common ST type was ST90. Among all of the isolates, a high ESBL/carbapenemases/plasmid AmpC (32.4%) prevalence was observed. A significant proportion of the isolates (37%) were members of high-risk clones including two pan-drug-resistant *K. pneumoniae* ST11 NDM-1 producing isolates. Due to extensive antibiotic usage during COVID-19, the situation may worsen, so routine screenings and strict infection control measures should be widely implemented.

## 1. Introduction

β-lactams are commonly used antimicrobials due to their safe profile and broad-spectrum activity [1,2,3,4,5]. β-lactamases are enzymes that can hydrolyze the β-lactams [1]. The most important β-lactamases are the extended-spectrum β-lactamases (ESBLs) and carbapenemases [2,3]. ESBLs are classified into three main groups—TEM, SHV, and CTX-M [1,4]. At the end of the previous century, ESBLs were mostly associated with TEM and SHV enzymes and with *Klebsiella pneumoniae* and hospital-acquired infections [4,5,6]. In the last two decades, the situation has changed. CTX-M ESBLs, mainly CTX-M-15 and CTX-M-14 were the most widespread variants among many *Enterobacterales* species [1,4,7]. The prevalence of ESBL producers has also increased in the community and among members of intestinal microbiota, mainly enteric bacteria. Extensive usage of carbapenems for treatment of infections caused by ESBL producers has led to an increase in the number of carbapenemase producers such as Klebsiella producing carbapenemase (KPC), New Delhi metalo-β-lactamase (NDM), and Verona integron-encoded metalo-β-lactamase (VIM) [2,5,6]. These bacteria are important causes of nosocomial and community-acquired infections [1,4,5,6]. Increasing rates of antibiotic resistance, especially in *K. pneumoniae*, *Escherichia coli*, and *Enterobacter cloacae* complex have been globally reported [1,4,6]. In addition, the increase was associated with dissemination of particularly high-risk clones that carry specific β-lactamases (ESBL or/and carbapenemases) [3,7]. These clones are defined as strains with a global distribution, with potential to spread, colonize, and persist in different niches and to acquire and exchange resistance determinants [7]. Many mobile elements (plasmids and integrons) contribute to the global dissemination of ESBLs or/and carbapenemases [4,7,8]. In addition, they carry genes, encoding nonsusceptibility not only to β-lactams, but also to many other antibiotic groups such as quinolones, aminoglycosides, tetracyclines, and trimethoprim/sulfamethoxazole [1,2,3,4,7,8].

The gastrointestinal tract can be an important reservoir of ESBL-/carbapenemase-producing enterobacteria in hospital settings [9] leading to further distribution of resistance determinants. Selective pressure of increased antibiotic usage and the dissemination of specific, often high-risk nosocomial clones additionally worsen the problem and could result in increased ESBL/carbapenemases gut carriage [8]. Such colonization among hospitalized patients can be a significant risk factor for different types of infections such as bloodstream, urinary tract, respiratory tract, surgical site, and abdominal infections; moreover, colonization with ESBL-/carbapenemases-producing enterobacteria could last for years [9,10,11].

Fecal carriage of ESBL/carbapenemase producers has been widely reported all over the world [9,10]. Differences in ESBL/carbapenemase fecal carriage rates have been reported, suggesting dynamics of their geographical evolution [9,10,11]. Data for fecal carriage of ESBL-/carbapenemase-producing enterobacteria in Bulgaria are limited. No multicenter investigations have been performed so far.

The aim of this study was to evaluate the prevalence of β-lactamase production and clonal relatedness of fecal ESBL- and carbapenemase-producing enterobacteria, collected from hospitalized patients (with >48 h hospital stay).

## 2. Materials and Methods

### 2.1. Bacterial Isolates-Collection and Isolation

The study was conducted in six hospitals—University Multiprofile Hospital for active treatment (UMHAT), Varna; UMHAT, Plovdiv; UMHAT, Pleven, and three hospitals in Sofia during the period of December 2017–June 2019. The fecal samples were obtained from patients (>48 h hospitalization) during the routine diagnostic process and were additionally inoculated on selective MacConkey agar with 1 mg/L cefotaxime and on Chromagar^TM^KPC (Becton Dickinson, Springfield, IL, USA). Bacterial isolates were identified using routine biochemical identification and were confirmed by VITEK (bioMérieux, Salt Lake City, UT, USA) or Phoenix (Becton Dickinson, Springfield, IL, USA). *Hsp60* sequencing [12] was used for species identification of *Enterobacter* spp. and *Klebsiella oxytoca* isolates.

### 2.2. Antimicrobial Susceptibility Testing

Antimicrobial susceptibility testing was performed using the disk diffusion method on Müller-Hinton II agar and the microdilution broth method according to the European Committee on Antimicrobial Susceptibility Testing (EUCAST) guidelines (Version 10). The susceptibility testing was carried out according to the clinical breakpoints of the EUCAST (Version 10) (http://www.eucast.org/clinical_breakpoints/), last accessed on 10 August 2022. The following antibiotics were tested: amoxicillin/clavulanic acid 30 µg (AMC), ceftazidime 10 µg (CAZ), cefotaxime 5 µg (CTX), cefepime 30 µg (FEP), cefoxitin 30 µg (FOX), imipenem 10 µg (IMP), meropenem 10 µg (MEM), piperacillin/tazobactam 36 µg (PIP/TAZ), chloramphenicol 30 µg (CHL), gentamicin 10 µg (GEN), amikacin 30 µg (AMK), tobramycin 10 µg (TOB), trimethoprim/sulfamethoxazole 25 µg (SXT), ciprofloxacin 5 µg (CIP), levofloxacin 5 µg (LVX), fosfomycin 200 µg (FOS)(for *E. coli* isolates), and tigecycline 15 µg (TIG for *E. coli* isolates). Susceptibility to tigecycline in *Klebsiella* isolates was determined by the broth microdilution method (Liofilchem, Italy). Susceptibility to colistin was initially tested with modified SuperPolymyxin medium [13]. Isolates of *K. pneumoniae* and *E. coli* that grew on this screening medium were tested with the broth microdilution method (Sensitest colistin, Liofilchem, Italy).

### 2.3. Phenotypic ESBL and Carbapenemases Detection

Presumptive ESBL production was detected with the double-disk synergy method [14]. Potential inducible AmpC producers were detected on the basis of antagonism between disks ceftazidime or cefotaxime and amoxicillin/clavulanic acid. In case of nonsusceptibility to carbapenems or/and growth on selective Chromagar^TM^KPC media (CHROMagar, Paris, France), a phenotypic confirmation of carbapenemase production was performed by the KPC, MBL(Metallo-β-lactamase), and OXA-48 Kit (Liofilchem, Roseto degli Abruzzi, Italy).

### 2.4. Isoelectric Focusing and Bioassay

Production and number of β-lactamases were detected and analyzed by analytical isoelectric focusing (IEF) according to the method of Mathew [15] with modifications [16] with Multifor II aparatus (Amersham Biosciences, Freiburg, Germany). The immobilized pH gradient polyacrylamid gel was made with ampholyte Pharmalyte 3–10 (Amersham Biosciences, Freiburg, Germany). β-lactamases were separated on the basis of their pH in the specific isoelectric points (pI). Nitrocefin (500 mg/L) was used to stain the β-lactamase’s bands. The hydrolytic activity of individual β-lactamase bands after the isoelectric focusing was assessed by a bioassay as previously described [16]. Two consecutive agar overlays were loaded onto the gel: the first overlay containing the respective β-lactam (cefotaxime 2 mg/L or imipenem 0.5 mg/L) and following 2 h incubation at 35–37 °C, by a second agar overlay containing the susceptible indicator strain *E. coli* K12:W3110 RifR lac/-/(1.2 × 10^7^ CFU/mL). After overnight incubation at 37 °C, growth of the indicator strain on the gel localized the β-lactamase band by which the β-lactam had been inactivated.

### 2.5. Molecular-Genetic β-lactamase Identification

All isolates were screened for the presence of carbapenemase-encoding genes (*bla*_VIM_, *bla*_IMP_, *bla*_KPC_, *bla*_NDM_, *bla*_OXA-48_), AmpC (*bla*_DHA_, *bla*_FOX_, *bla*_MOX_, *bla*_CMY_, *bla*_ACC_), and ESBLs *(bla*_CTX-M_, *bla*_SHV_, *bla*_TEM_) as previously described [16,17,18,19]. The genes were sequenced using primers binding outside the coding region of *bla*_SHV_, *bla*_CTX-M-1-_group, *bla*_CTX-M-9-_group, *bla*_CMY,_ *bla*_DHA,_ *bla*_KPC_, and *bla*_NDM_. [16,20]. Nucleotide and deduced amino acid sequences were analyzed and multiple alignments were performed using Chromas Lite 2.01 (Technelysium Pty Ltd., Brisbane, Australia) and DNAMAN version 8.0 software (Lynnon BioSoft, Vaudreuil-Dorion, GM, Canada).

### 2.6. ERIC, MLST Typing, and Phylotyping

Clonal relatedness was investigated by ERIC PCR and Multilocus Sequence Typing (MLST). For Enterobacterial Repetitive Intergenic Consensus (ERIC)-PCR, ERIC-1 and -2a primers were used as described previously [21]. Genetic similarity was determined using Dice coefficient as similarity measure and the unweighted pair group method with arithmetic mean (UPGMA) (http://genomes.urv.cat/UPGMA/), last accessed on 30 July 2022. A clone was defined as isolates showing 80% similarity.

Pasteur scheme was used for *K. pneumoniae* MLST typing and Achtman scheme was applied for *E. coli*. For *E. coli* isolates, the assignment to allelic numbers and sequence types (STs) was performed according to the MLST database (https://bigsdb.web.pasteur.fr/ecoli/ecoli.html), last accessed on 30 July 2022. Detection of specific O25b-ST131 clone was performed with allele-specific PCR for *pabB* gene as previously described [22].

For *K. pneumoniae* isolates, protocols and assignment to allelic numbers and sequence-types (STs) were carried out as described in the MLST database (Pasteur Institute, Paris, France; http://bigsdb.web.pasteur.fr/klebsiella/klebsiella.html), last accessed on 30 July 2022. A clonal complex was defined as a group of two or more independent isolates that shared six identical alleles.

For *E. cloacae* complex isolates, primers, protocols, and assignment to allelic numbers and sequence-types (STs) were carried out as described in the MLST database (https://pubmlst.org/organisms/enterobacter-cloacae), last accessed on 30 July 2022.

Phylotyping was applied for *E. coli* isolates as previously described [23].

## 3. Results

### 3.1. Bacterial Isolates

A total of 205 enterobacterial isolates resistant to cefotaxime were isolated from fecal samples, collected from 580 patients (35.3%) as follows: 61 cefotaxime-resistant isolates from 158 studied patients (38.6%) from University Hospital (UH)-Varna; 25 cefotaxime-resistant isolates from 71 patients (35.2%) from UH-Pleven; 58 cefotaxime-resistant isolates from 102 patients (56.9%) from UH-Plovdiv, and 61 cefotaxime-resistant isolates from 249 patients (24.5%) from three hospitals in Sofia. The isolates were identified as *E. coli* (*n* = 103); *K. pneumoniae* (*n* = 65); *Klebsiella oxytoca* (*n* = 8); *E. cloacae complex* (*n* = 16); *Morganella morganii* (*n* = 3); and *Citrobacter freundii complex* (*n* = 10).

*Hsp60* sequencing identified 15 *E. cloacae* complex isolates as *Enterobacter hormaechei*, subdividing them into three subspecies—*E. hormaechei* spp. *hoffmannii* (*n* = 3); *E. hormaechei* spp. *steigerwaltii* (*n* = 10); and *E. hormaechei* spp. *xiangfangensis* (*n* = 2). One isolate was identified as *Enterobacter kobei*. Two isolates, initially identified as *K. oxytoca*, were reidentified by *hsp60* sequencing as *Klebsiella michiganensis*.

### 3.2. Antimicrobial Susceptibility Testing

The nonsusceptibility (resistance or intermediate susceptibility) rates in the collection of isolates were as follows: amoxicillin/clavulanic acid 93%, ceftazidime 86%, cefotaxime 100%, cefoxitin 35%, cefepime 95%, piperacillin/tazobactam 72%, imipenem 7%, meropenem 7%, tobramycin 68%, gentamicin 56%, amikacin 50%, trimethoprim/sulfamethoxazole 52%, ciprofloxacin 68%, levofloxacin 63%, and chloramphenicol 22%. The nonsusceptibility rates in the *Klebsiella* isolates to tigecycline was 93%. For *E. coli*, 17% and 3% resistance to tigecycline and fosfomycin, respectively, were found. Only five isolates of *Klebsiella* spp. (2.4%) were colistin nonsusceptible. The results are shown in Table S1. Two *K. pneumoniae* isolates were determined as pandrug-resistant according to the criteria of Magiorakos et al. [24].

### 3.3. Phenotypic ESBL and Carbapenemase Detection

Disk diffusion synergy method (DDST) confirmed 171 isolates (171/205, 83.4%) as ESBL producers. Four *E. cloacae* complex and three *C. freundii* complex isolates were determined as possible inducible AmpC hyperproducers, demonstrating antagonism between amoxicillin/clavulanic acid and cefotaxime or ceftazidime.

The phenotypic test with meropenem and meropenem/EDTA disks was positive in 11 carbapenem-resistant *K. pneumoniae* isolates, suggesting class B carbapenemase production (zone around the disk with EDTA is ≥5 mm). Three isolates (two *K. pneumoniae* and one *E. coli*) demonstrated increased zones of inhibition by the disk containing meropenem and phenylboronic acid, suggesting class A (KPC) carbapenemase activity.

### 3.4. Molecular-Genetic Identification of β-lactamase

PCR and sequencing revealed the presence of genes, encoding ESBL in 167 (28.8%) isolates, obtained from 580 patients. In one isolate, only *bla*_SHV-1_ gene was detected (which suggests possible SHV-1 hyperproduction). Three cefotaxime-resistant isolates, susceptible to carbapenems and with positive DDST did not produce a positive PCR reaction with any of the used ESBL group-specific primers.

The prevalence of ESBL producers in Sofia hospitals was significantly lower (19.7%, 49 ESBL producers from 249 patients) than in the other locations, 35.6% (118 ESBL producers/331 patients) (*p* < 0.0001). The ESBL producers among the isolates of *E. coli* and *K. pneumoniae* were 56.2% (94/167) and 34.1% (57/167), respectively.

Among the 205 investigated cefotaxime-resistant isolates, we observed solely *bla*_CTX-M-15_ in 41% (84/205) of the isolates, *bla*_CTX-M-3_ in 24% (49/205), *bla*_CTX-M-27_ in 11% (22/205), and 8 isolates (3.9%) displayed *bla*_CTX-M-14_. Only one isolate was positive for *bla*_CTX-M-9_ and one for *bla*_SHV-12_ (Table 1). *bla*_CTX-M-15_ was more often detected among *E. coli*, *E. hormachei, C. freundii* complex, and *M. morganii* isolates. Almost all members of CTX-M-9 family (*bla*_CTX-M-9,-14,-27_), except one isolate that showed *bla*_CTX-M-14_, were produced by *E. coli* strains. In contrast, *bla*_CTX-M-3_ was the prevailing ESBL among *K. pneumoniae* and *K. michiganensis* isolates. One *K. pneumoniae* isolate showed mixed sequences (GA/GT, both A and G in a codon at position 238, GAT is a codon for aspartic acid (specific for *bla*_CTX-M-3_), GGT is a codon for glycine (specific for *bla*_CTX-M-15_)). All *K. pneumoniae* isolates were *bla*_SHV_ positive. Of them, 29 representative isolates were sequenced, and *bla*_SHV-1_ (*n* = 21) and *bla*_SHV-11_ (*n* = 8) were identified. In 71 isolates, *bla*_TEM_ was found and, later, 10 of them were confirmed by sequencing as *bla*_TEM-1_.

Fourteen isolates (2.4%) from 580 patients, mainly *K. pneumoniae,* were carbapenemase producers. Thirteen of them coproduced ESBL or/and plasmid AmpC (Table 1). Ten *K. pneumoniae* isolates had *bla*_NDM-1_ together with *bla*_CMY-4_ and *bla*_CTX-M-3/-15_. Two *K. pneumoniae* and one *E. coli* isolates had *bla*_KPC-2_.

Seven *E. coli* (1.2%, 7/580) isolates showed two types of plasmid AmpC, *bla*_DHA-1_, *n* = 5 or *bla*_CMY-2_, *n* = 2. Thirteen isolates (2.2%) were AmpC hyperproducers (inducible or constitutive) (Table 1).

Thus, 188 isolates (32.4%) were found to carry ESBL (28.8%) or carbapenemases (2.4%) or plasmid AmpC genes (1.2%).

### 3.5. Isoelectric Focusing and Bioassay

The isoelectric focusing was carried out in representative isolates according to the species and detected β-lactamase genes. Of 73 *Klebsiella* spp. isolates, 24 representative isolates were subjected to IEF (12 *bla*_CTX-M-3_ positive, 4 *bla*_CTX-M-15_ positive, the isolate with mixed sequence, 4 *bla*_NDM-1_, and all the *bla*_KPC-2_ isolates) (Table S2). We confirmed the production of CTX-M-15 (observed band with pI8.8 (β-lactamase, focused at isoelectric point (pI) with pH 8.8)), CTX-M-3 (band with pI8.4). Two *bla*_KPC-2_ isolates had bands at pI6.7 corresponding with KPC-2. *bla*_NDM-1/CMY-4/CTX-M-15/-3_ positive isolates produced two bands—one at pI9.2 corresponding with CMY-4 and the other at pI8.8 or pI8.4—CTX-M-15 or CTX-M-3. NDM-1 enzyme was not visualized. The isolate for which we supposed a coproduction of two CTX-M enzymes (with mixed sequence) exhibited two bands—pI8.4 and pI8.8 (Figure S1) (CTX-M-3-like and CTX-M-15-like, respectively).

From 103 *E. coli* isolates, we tested 28 representative ones. Four *bla*_CTX-M-3_ positive, nine *bla*_CTX-M-15_, eight *bla*_CTX-M-27_, one *bla*_CTX-M-14_, and a single *bla*_CTX-M-9_ isolate gave cefotaxime-hydrolyzing bands at pI8.4, pI8.8, pI8.2, and pI8.1, pI8.1, respectively (Table S2). IEF revealed the presence of bands with pI7.8 (three isolates) and >8.8 (two isolates) corresponding with DHA-1 and CMY-2 enzymes, respectively.

All tested isolates showed only one type of ESBL or/and carbapenemases (have only one CTX or IMP hydrolyzing band) and did not show cefotaxime hydrolyzing bands corresponding to TEM and SHV ESBL β-lactamases. Three isolates with unidentified enzyme type gave bands with pI8.0 (two isolates) and pI9.2 (one isolate). They were without CTX hydrolytic activity.

Among 16 ESBL positive *E. hormaechi*, *C. freundii* complex and *M. morganii* isolates, 5 isolates were tested, and a single cefotaxime hydrolyzing band was detected, pIs corresponded with sequenced enzymes (Table S2).

### 3.6. MLST, ERIC Typing, and Phylotyping

Epidemiological typing revealed 18 ERIC clusters (Figure S2) among 65 *K. pneumoniae* isolates, corresponding to 17 ST types, with ST353 (*n* = 12, 19%), ST11 (*n* = 11, 16.9%), and ST37(*n* = 8, 12%) being the dominant types. In addition, ST1198, ST280, ST34, ST15, ST258, ST17, ST253, and ST449 were also found. Six isolates demonstrated unique profiles (Table 2). The isolates from the prevalent ST353 were *bla*_CTX-M-3_ positive and were isolated mainly from patients hospitalized in the Plovdiv University hospital (Table 2). ST11 isolates were *bla*_NDM-1_ positive. *bla*_KPC-2_ carbapenemase was detected in ST258 isolates. Both of them were collected from hospitals in Sofia. The isolates from high risk clones ST11, ST258, and ST15 were 26% (*n* = 17 from 65 *K. pneumoniae* isolates). *K. oxytoca* and *K. michiganensis* isolates had unique ERIC profiles.

Among *E. coli* isolates, 40 ERIC types were identified (Figure S3). A total of 21 isolates exhibited unique ERIC profiles and the other 19 had between 2 and 43 members. MLST defined six main ST types: ST131 (*n* = 43, 41.7%), ST38 (*n* = 10, 9.7%), ST155 (*n* = 5, 4.9%), ST405 (*n* = 3, 2.9%), ST1196(*n* = 4, 3.9%), and ST88 (*n* = 3, 2.9%).

ST131 *E. coli* (*n* = 43) was detected in five centers and was associated with the production of CTX-M-27 (46.5%, 20/43), CTX-M-15 (39.5%, 17/43), CTX-M-3 (7%, 3/43), CTX-M-14 (5%, 2/43), and KPC-2 carbapenemase, found in a single isolate (Table 3 and Table S2). Twenty-six of all ST131 isolates (60.5%) were *pabB* positive, belonging to the highly virulent O25b:H4-ST131. Most of the non-O25b isolates produced CTX-M-27 enzymes.

The following *E. coli* phylotypes were identified: B2, *n* = 51 (49.5%, 51/103); D, *n* = 20 (19.4%, 20/103); A, *n* = 12; and B1, *n* = 20. The association between MLST types and phylotypes is shown in Table 3. The B2 group included isolates from ST131, ST95, ST127, ST144, and ST681; D group—isolates from ST38 and ST405, ST69; A group—clonal complex (CC)10 and CC23; and B1—CC155 and CC648, respectively.

*E. cloacae* isolates (*n* = 16) demonstrated 12 ERIC clusters and 6 ST types. The most common ST was ST90, associated with CTX-M-15 production (Table 4).

## 4. Discussion

This study reveals a moderate rate of fecal colonization with ESBL producers (28.8%) among Bulgarian patients during the period 2017–2019. The rate of third-generation cephalosporin-resistant isolates (mostly due to production of ESBL/carbapenemase/plasmid AmpC) was high (35.3%), but lower than the rate observed in a pilot study on hospital fecal carriage in Varna city, Bulgaria in 2015 (42.5%) [25]. A possible reason could be the multicenter design of the present study. It represents the β-lactamase fecal carriage among Bulgarian patients, hospitalized in three centers in the capital city of Sofia and three other hospitals in main Bulgarian cities (Varna, Plovdiv, and Pleven). In addition, in this study, the rate of ESBL-producing isolates among hospitalized patients in Sofia was significantly lower compared with that in patients from the other centers. The ESBL fecal prevalence in the whole patient group (*n* = 580) was similar to that in Portuguese hospitals (24%) [26] and higher than that in French hospitals (17.7%) [27]. The observed frequency in our study was much lower than the rate reported from Africa and Asia (>50% in China, Chad, and Egypt) [28]. Our findings are in concordance with the high incidence of third-generation cephalosporin-resistant invasive *E. coli* and *K. pneumoniae* isolates (38.6% and 75.7%, respectively) in Bulgaria in 2019, which was the highest in Europe and showed a stable trend during the last 5 years (https://www.ecdc.europa.eu/sites/default/files/documents/Additional-tables-EUEEApopulation-weighted-mean-2019.pdf), last accessed on 10 August 2022. We also found increased nonsusceptibility rates in the tested ESBL fecal isolates to aminoglycosides (50%-68%) and quinolones (63–68%) in comparison to the rates found in 2015 (21%-57% for aminoglycosides and 15–27% for quinolones) [25]. A similar trend was detected for carbapenem resistance—7% during the period of 2017–2019 and 3% in 2015 [25].

In comparison with our previous study in 2015, a decreased rate of ESBL-producing *E. coli* (56.2% versus 69%) and an increased proportion of ESBL *K. pneumoniae* isolates (34.1% versus 20.4%) were found [25]. Similar results were reported from Portugal [26]. The predominant levels of ESBL-producing fecal isolates *E. coli* have been reported in many studies [9,10,26,27,28].

The main β-lactamase type in our study was CTX-M-15 (41%). It was the predominant ESBL in *E. coli*, as well as in all other species except *Klebsiella* spp. CTX-M-15-producing isolates have been reported worldwide [1,2,4,5,6,8,9]. CTX-M-3 β-lactamase was the second most common ESBL (24%) in the current study. This enzyme is produced mainly by *Klebsiella* isolates. Similar to CTX-M-15, CTX-M-3 was also widely distributed among enteric strains [9,10,26,27,28]. The proportion of CTX-M-9 group ESBLs was lower (15.4%) and presented by CTX-M-27 (11%), CTX-M-14 (3.9%), and CTX-M-9 (0.5%). These enzymes were predominantly identified among *E. coli* isolates. CTX-M-9 and CTX-M-14 intestinal producers have been reported in Portugal, Spain, China, and Bulgaria [9,25,26]. CTX-M-27 was associated with fecal carriage isolates in a Portuguese study [26]. In Bulgaria, it has been reported for the first time. The plasmid AmpC enzymes DHA-1 and CMY-2 in the present study were produced by *E. coli* isolates. In Bulgaria, DHA-1 enzyme was identified for the first time in 2019 in an *E. cloacae* complex isolate obtained from a blood sample [29].

An important finding in the current study is the detection of carbapenemase-producing isolates. Although the detection rate was low (2.4%), it is an indicator that the enteric tract may act as a reservoir for these problematic bacteria. The carbapenem resistance in the present collection of fecal isolates was associated with NDM-1 and KPC-2, detected in *K*. *pneumoniae* isolates and in a single *E. coli* isolate. All carbapenem-producing isolates were obtained from hospitalized patients in Sofia. Interestingly, the frequency of ESBL producers (19.7%) was not high among these patients. The coproduction of NDM-1, CTX-M-15/-3, and CMY-4 enzymes in nine *K. pneumoniae* isolates is a possible explanation for the high resistance rates, identified in the NDM-1 producing isolates, two of them being pandrug-resistant.

The rates of carbapenemase producers in Europe have steadily increased during the last years in both clinical and intestinal isolates [2,30,31,32,33,34,35]. The fecal colonization with carbapenemase producers is of high clinical importance because of its possible prolonged persistence over time (387 days) [36].

Another important finding in the present study is the identification of the high-risk clones ST11, ST258, and ST15 (*n* = 17, 26%) among *K. pneumoniae* fecal isolates. Taking the second place, ST11 was represented in 17% of *K. pneumoniae* isolates, all obtained from patients in Sofia hospitals. Most NDM-1 carbapenemase-producers belonged to this clone, including two panresistant and the colistin-resistant isolates. This result is in concordance with our previous study from 2019 that detected a high level of ST11 *bla*_NDM-1_/*bla*_CTX-M-15 or-3_/*bla*_CMY-4_ positive isolates in the same hospitals [21]. ST11 is one of the most widely distributed clones, prevalent among clinical isolates in Europe (Portugal, Poland, Greece), Asia, and particularly in China [2,30,32,35,37,38,39,40]. The ST11 *bla*_NDM-1_/*bla*_CTX-M-15/3_/*bla*_CMY-4_ positive isolates have also been detected in the Czech Republic [39]. We can assume that the coproduction of three different β-lactamases (carbapenemase, AmpC, and ESBL) in these isolates contributes to ST11 sustainable distribution, making the patients’ intestinal tract a reservoir of extremely difficult to treat bacteria. The ST11 clone, producing various carbapenemases (NDM-1, NDM-5, KPC-2, and OXA-48), was identified not only among clinical [30,32,33,37] but also among fecal isolates [35,40,41]. Increased antibiotic pressure, especially during the COVID-19 pandemic, could promote their dissemination.

ST258 *K. pneumoniae*, a single locus variant of ST11 (*tonB*), is another high-risk clone. It is also distributed worldwide and associated with KPC-2 production [2,6,30,33,41]. In this study, only single ST258 isolates were detected. This is in concordance with the results of our previous study from 2019 [21]. ST11 and ST258 clones easily obtain different plasmids and other mobile genetic elements and are good promoters of drug resistance. [2,3,4,6,7,33,35,38,40,42,43].

ST15 is another high-risk *K. pneumoniae* clone which has commonly been associated with the production of ESBLs, mainly CTX-M-15 [3,7,44], but also is involved in KPC dissemination [30]. In the present study, only four carbapenem-susceptible isolates were identified as ST15 producing CTX-M-15 ESBL, confirming the results in Bulgaria in the last years [25].

Other intestinal multidrug-resistant *K. pneumoniae* clones found in this study were ST37 and ST17. These clones showed a sustained persistence in Bulgaria. These ST have been already identified in clinical and fecal isolates, associated with CTX-M-15 and KPC-2 production [7,26,45]. ST37 and ST17 have been reported as important worldwide distributed MDR clones. Moreover, they can easily acquire mobile elements that carry carbapenemase genes, providing them an additional advantage [3,7,30].

In the present study, ST353 was presented by a high number of isolates (19%), mainly obtained from patients hospitalized in Plovdiv University Hospital(A). ST353 isolates were associated mainly with CTX-M-3 production. This ST type has been rarely reported. In studies from China and Colombia, authors reported ST353 isolates, producing KPC and OXA-48 [46,47]. ST1198 is also a rare ST. Interestingly, both ST types showed relatedness to ST37, (two-allele difference). A further investigation of ST353 is needed, because of its possible potential to be a high-risk clone.

Five high-risk clones were observed among *E. coli* isolates with ST131 being the predominant, found in 41.7%. The second most common ST was ST38 (10%). ST405, ST95, and ST69 were presented by single isolates only. The isolates from these high-risk clones represented 57% (*n* = 59) of all the studied *E. coli* isolates. All of them belonged to the B2 or D phylogroups. This is an important finding as these phylogroups are associated with a prolonged fecal carriage [48]. This will increase the possibility for members of the high-risk clones to cause both community- and healthcare-associated infections such as urinary tract and bloodstream infections.

Almost all ST131 isolates (*n* = 43) were representatives of phylogroup B2 and 60% (*n* = 26) belonged to the O25b:H4-ST131 clone. Our study showed that the O25b:H4-ST131 clone was the major clone among fecal *E. coli* isolates, harboring different ESBL genes, mainly *bla*_CTX-M-15_, but also *bla*_CTX-M-3_ and *bla*_CTX-M-14_, which is in concordance with other reports [49]. Only one isolate, belonging to O25b:H4-ST131, produced KPC-2 carbapenemase. O25bST131 clone is a highly virulent, pandemic clone [49,50,51]. Its widespread dissemination has been associated with some urological procedures, increased antibiotic usage [4], and the enhanced ability to colonize the intestinal tract [51]. The non-O25b ST131 isolates in this study predominantly produced CTX-M-27. In recent years, representatives of ST131 producing CTX-M-27 have been increasingly detected in Asia, Australia, Canada, USA, and Europe [4,6,26,52,53]. Other authors reported that CTX-M-27 producing ST131 isolates spread more efficiently than CTX-M-15 ST131 [54]. ST131 *E. coli* isolates were detected in all centers except the Sofia hospital 1 (D), where NDM-1-producing ST11 *K. pneumoniae* was observed. In addition, the frequency of ST131 isolates in the present study was higher (42%) than the rate (35%) detected in 2015 [25]. This result is a concern as ST131 isolates have been associated with prolonged fecal carriage [52].

Our results showed that the gut could be an important reservoir of highly resistant and virulent bacteria in hospitals. This could increase the incidence of infection, as some reports showed high similarity between fecal isolates and clinical isolates: the report was for transplant patients [55]. In the time of increased antimicrobial usage during the COVID-19 pandemic, an increased frequency of such bacteria could be expected, as the previous antibiotic treatment is one of the most important factors for ST131 dissemination [4,49]. Regular follow-up studies are of great importance.

In our study, *E. coli* ST38, belonging to D phylogroup, was the second most commonly isolated type, associated with production of a wide range of ESBLs (CTX-M-15, CTX-M-3, CTX-M-14, and CTX-M-27) and the plasmid AmpC enzyme DHA-1. This type has also been reported as a high-risk clone, causing both nosocomial and community-acquired infections, mainly urinary tract infections [4,7]. Another high-risk clone identified in this study is ST95, presented as a single CTX-M-14 producing isolate, also belonging to the virulent B2 phylogroup. The representatives of ST95 have demonstrated an increased virulence and have often caused urinary and bloodstream infection [51]. The detection of ST95 is an indicator for its stable persistence in Bulgarian patients as it was identified in fecal isolates in 2015 [25].

ST69 is an interesting lineage from the D phylogroup, identified in two isolates of *E. coli*. Recently, it has been found that representatives of ST69 clone carry an intact locus of enterocytes effacement (LEE), coding second bacterial type III secretion systems involved in the pathogenesis of Gram-negative infections [56]. In our study, three CTX-M-15 producing isolates of *E. coli* were identified as ST405 (D phylogroup). This clone was previously detected in Bulgaria as a carrier of NDM-1 carbapenemases [57]. *E. coli* ST405 is an emerging urosepsis pathogen, reported to carry *bla*_CTX-M_, *bla*_NDM_, and a number of virulent genes comparable with O25b:H4-ST131 [58].

In addition to the isolates that represent highly virulent clones, we also detected *E. coli* isolates belonging to B1 and A phylogroups, which have been reported as commensal gut bacteria [10]; the observed isolates belonged mostly to CC10 and CC155 (11 isolates).

In the group of *Enterobacter* isolates, ST90 producing CTX-M-15 was detected as the dominant clone. Although no high-risk clones have been defined in *Enterobacter* spp. so far, ST90 isolates, producing different carbapenemases, were detected in many countries: VIM-1 in Greece, IMP-4 in Canada and UK, and NDM-1 in Romania [59]. Polish authors reported a wide ST90 hospital dissemination [60]. So, we can assume that ST90 *E. cloacae* complex can be a candidate for an international high-risk clone.

The fecal colonization with ESBL and/or carbapenemase producers from high-risk international clones, associated with significant virulence and invasive potential and multidrug or pandrug resistance, can be an important reservoir not only for difficult to treat nosocomial infections, but also for wide dissemination in the community. Given that this study was performed before the COVID-19 pandemic, we can assume that the increased antibiotic usage during the last three years has further worsened the situation.

## 5. Conclusions

In conclusion, a high rate of fecal colonization (32.3%) with ESBL/carbapenemase/plasmid AmpC producers among patients in Bulgarian hospitals was found. A high rate of ESBL producers (28.8%) was detected with a relatively low rate of carbapenemase producers (2.4%). Seventy six isolates (59 *E. coli* and 17 *K. pneumoniae*) were members of high risk clones (37%).Twenty six percent of the *K. pneumoniae* isolates were representatives of high-risk clones such as ST11, ST258 and ST15 *K. pneumoniae* isolates. A very worrying finding was the detection of two ST11 pandrug resistant isolates, coproducing NDM-1, CMY-4, and CTX-M-15. Among *E. coli*, five high risk clones (57% of *E. coli* isolates) were found (ST131, ST38, ST405, ST69, and ST95). The investigated isolates were producers of a wide range of β-lactamases—CTX-M-15, CTX-M-3, CTX-M-27 (reported for the first time in Bulgaria), CTX-M-14, NDM-1, KPC-2, and plasmid AmpC DHA-1 and CMY-2 enzymes. The detected high frequency of ESBL/carbapenemases-producing enteric bacteria before COVID-19 and the dramatically increased selective pressure during the pandemic period will negatively impact the antimicrobial resistance in clinically significant bacterial species. Further studies should closely monitor the future trends. The routine screening for colonization with MDR bacteria at hospital admission and during the hospital stay, especially in high-risk departments, as well as strict infection control measures should be widely implemented in Bulgarian hospitals to limit the further dissemination of problematic multidrug-resistant bacteria.

## Figures and Tables

**Table 1:** Distribution of ESBLs, carbapenemases, and AmpC enzymes according to bacterial species.

Bacterial Species β-lactamase Genes Detected	*E.coli* *n* = 103	*Klebsiella* spp. *n* = 73	*Enterobacter* spp. *n* = 16	*C. freundii* Complex *n* = 10	*M. morganii* *n* = 3	Total Number *n* = 205
*bla*_CTX-M-15_	46 (45%)	24 (33%)	7 (44%)	6 (60%)	1 (33%)	84 (41%)
*bla*_CTX-M-15_ + *bla_C_*_MY-4_	-	1 (1.4%)	-	-	-	1 (0.5%)
*bla*_CTX-M-3 like_ + *bla*_CTX-M-15 like_	-	1 (1.4%)	-	-	-	1 (0.5%)
*bla*_CTX-M-3_	18 (17%)	30 (41%)	1 (6%)	-	-	49 (24%)
*bla*_CTX-M-9_	1 (1%)	-	-	-	-	1 (0.5%)
*bla*_CTX-M-14_	7 (7%)	1 (1.4%)	-	-	-	8 (3.9%)
*bla*_CTX-M-27_	22 (21%)	-	-	-	-	22 (11%)
*bla*_SHV-12_	-	-	-	1 (10%)	-	1 (0.5%)
*bla*_NDM-1_ + *bla*_CTX-M-15_ + *bla_C_*_MY-4_	-	9 (12%)	-	-	-	9 (4.4%)
*bla*_NDM-1_+ *bla*_CTX-M-3_ + *bla_C_*_MY-4_	-	1 (1.4%)	-	-	-	1 (0.5%)
*bla*_NDM-1_+ *bla*_CTX-M-3_	-	1 (1.4%)	-	-	-	1 (0.5%)
*bla*_KPC-2_	-	1 (1.4%)	-	-	-	1 (0.5%)
*bla*_KPC-2_ + *bla*_CTX-M-15_	1 (1%)	-	-	-	-	1 (0.5%)
*bla*_KPC-2_+ *bla*_CTX-M-3_	-	1 (1.4%)	-	-	-	1 (0.5%)
*bla*_DHA-1_	5 (5%)	-	-	-	-	5 (2.4%)
*bla*_CMY-2_	2 (2%)	-	-	-	-	2 (1%)
Other *	-	1 (1.4%)	-	-	-	1 (0.5%)
Unknown mechanism	1 (1%)	2 (3%)	-	-	-	3 (1.5%)
AmpC hyperproducers	-	-	8 (50%)	3 (30%)	2 (67%)	13 (6%)

* hyperproduction of SHV-1 enzyme.

**Table 2:** Distribution of MLST types in 65 cefotaxime-resistant *K. pneumoniae* isolates according to the center and the β-lactamase production.

ERIC TYPE (Number)	MLST Type (Number Isolates)	Center (Number Isolates)	Detected Gene/Gene Combinations (Number)
b (*n* = 10) b”(*n* = 2)	ST353 (*n* = 12)	A (11); D (1)	*bla*_CTX-M-3_ (10) unidentified (2)
p (*n* = 11)	ST11	D (11)	*bla*_NDM-1_ + *bla*_CTX-M-15_ + *bla_C_*_MY-4_ (9) *bla*_NDM-1_ + *bla*_CTX-M-3_ + *bla_C_*_MY-4_ (1) *bla*_NDM-1_ + *bla*_CTX-M-3_ (1)
s (*n* = 8)	ST37	A (2); B (6)	*bla*_CTX-M-3_ (6) *bla*_CTX-M-15_ (2)
a (*n* = 6)	ST1198	A (6)	*bla*_CTX-M-15_
h (*n* = 4)	ST280	B (4)	*bla*_CTX-M-15_ (3) *bla*_CTX-M-3_ (1)
w (*n* = 3)	ST34	B (1); E (2)	*bla*_CTX-M-15_ (2) *bla*_CTX-M-14_ (1)
m (*n* = 4)	ST15	B (1); E (3)	*bla*_CTX-M-15_
t (*n* = 3)	ST1569	E (1); A (2)	*bla*_CTX-M-3_ (2), *bla*_CTX-M-15_ (1)
c (*n* = 2)	ST17	A (2)	*bla*_CTX-M-3_ (1) *bla*_CTX-M-15_ (1)
n (*n* = 2)	ST258	D (1); F (1)	*bla*_KPC-2_ (1) *bla*_KPC-2_ + *bla*_CTX-M-3_ (1)
r (*n* = 2)	ST253	E (2)	*bla*_CTX-M-15_
e (*n* = 2)	ST449	C (1); F (1)	*bla*_CTX-M-3_ (1) *bla*_CTX-M-15_ + *bla_C_*_MY-4_ (1)
uni (*n* = 6)	ST429 (*n* = 1) ST627 (*n* = 1) ST20 (*n* = 1) ST215 (*n* = 1) ST1563 (*n* = 1) ND (*n* = 1)	A (1) F (1) A (1) B (1) F (1) B (1)	*bla*_CTX-M-3_ *bla*_SHV-1_ *bla*_CTX-M-3_ CTX-M-3/CTX-M-15 like *bla*_CTX-M-15_ *bla*_CTX-M-15_

Abbreviations: *n*—number of isolates; A—University hospital Plovdiv; B—University hospital Varna; C—University hospital Pleven; D—First Sofia hospital; E—Second Sofia hospital; F—Third Sofia hospital; uni—unique profile.

**Table 3:** Distribution of MLST types in 103 cefotaxime-resistant *E. coli* isolates according to the center and the β-lactamase production.

ERIC Type (Number)	MLST Type (Number)	CC	Phylo Group	Center (Number Isolates)	Detected Gene (Number)
A (*n* = 32) A1 (*n* = 1) A2 (*n* = 3) A3 (*n* = 1) A4 (*n* = 3) A5 (*n* = 3)	ST131(*n* = 43)	131	B2	A (3); B (11); E (6); C (14); F (9)	*bla*_CTX-M-15_ (17), *bla*_CTX-M-3_ (3) *bla*_CTX-M-27_ (20), *bla*_CTX-M-14_ (2), *bla*_KPC-2_ (1)
S1 (*n* = 4) S2 (*n* = 6)	ST38 (*n* = 10)	38	D	A (3); B (3) F (3); E (1)	*bla*_CTX-M-15_ (3), *bla*_CTX-M-3_ (1) *bla*_CTX-M-14_ (3), *bla*_CTX-M-27_ (2), *bla*_DHA-1_ (1)
X (*n* = 1)	ST4981	10	A	E (1)	unidentified (1)
F (*n* = 5)	ST155	155	B1	A (2); B (3)	*bla*_CTX-M-15_ (3), *bla*_CTX-M-3_ (2)
K (*n* = 2)	ST69	69	D	B (2)	*bla*_DHA-1_ (1), *bla*_CTX-M-3_ (1)
H (*n* = 4)	ST1196	-	B1	A (3)_;_ B (1)	*bla*_CTX-M-15_ (1), *bla*_CTX-M-3_ (2), *bla*_DHA-1_ (1)
W (*n* = 3)	ST405	405	D	B (2); F (1)	*bla*_CTX-M-15_ (3)
F1 (*n* = 2)	ST56	155	B1	B (2)	*bla*_CTX-M-3_ (1), DHA-1 (1)
J (*n* = 1)	ST10	10	A	A (1)	*bla*_CTX-M-3_ (1)
Z (*n* = 1)	ST88	23	A	A (3)	*bla*_CTX-M-3_ (2), *bla*_CTX-M-15_ (1)
R (*n* = 2)	ST1011	-	D	A (1); B (1)	*bla*_CTX-M-14_ (1), *bla*_CTX-M-9_ (1)
L (*n* = 2)	ST127	-	B2	A (2)	*bla*_CTX-M-15_ (2)
T (*n* = 2)	ST144	-	B2	A (2)	*bla*_CTX-M-15_ (2)
N (*n* = 2)	ST1485	648	B1	B (2)	*bla*_CTX-M-3_ (2)
V (*n* = 1)	ST34	10	A	A (1)	*bla*_CTX-M-15_ (1)
C (*n* = 2)	ST681	-	B2	B (2)	*bla*_CTX-M-3_ (1), *bla*_CTX-M-15_ (1)
B (*n* = 1)	ST95	95	B2	C (1)	*bla*_CTX-M-14_ (1)
D (*n* = 1)	ST1993	-	B2	A (1)	*bla*_CTX-M-15_ (1)
uni (*n* = 15)	ND		B1(7) A(5); D(3)	B (5), F (4) A (3); E (3)	*bla*_CTX-M-15_ (10), *bla*_CTX-M-3_ (2), *bla*_CMY-2_ (2), *bla*_DHA-1_ (1)

Abbreviations: *n*—number of isolates; A—University hospital Plovdiv; B—University hospital Varna; C—University hospital Pleven; D—First Sofia hospital; E—Second Sofia hospital; F—Third Sofia hospital; CC—clonal complex; uni—unique profile.

**Table 4:** Distribution of MLST types in 16 cefotaxime-resistant *E. cloacae* complex isolates according to the center and the β-lactamase production.

ERIC Type (Number)	Species	MLST	Center (Number)	Detected Gene (Number)
A (*n* = 5)	*E. hormaechei* spp. *steigerwaltii*	ST90	B (4), E (1)	*bla*_CTX-M-15_ (5)
V (*n* = 2)	*E. hormaechei* spp. *hoffmanii*	ST128	A (2)	AmpC (2)
uni (*n* = 1)	*E. hormaechei* spp. *hoffmanii*	ND	B (1)	*bla*_CTX-M-15_ (1)
uni (*n* = 1)	*E. hormaechei* spp. *hoffmanii*	ST104	C (1)	AmpC (1)
uni (*n* = 1)	*E. hormaechei* spp. *xiangfangensis*	ST148	F (1)	AmpC (1)
uni (*n* = 1)	*E. hormaechei* spp. *xiangfangensis*	ST200	F (1)	*bla*_CTX-M-3_ (1)
uni (*n* = 1)	*E. hormaechei* spp. *steigerwaltii*	ST1116	B (1)	AmpC (1)
uni (*n* = 1)	*E. hormaechei* spp. *steigerwaltii*	ND	C (1)	AmpC (1)
uni (*n* = 1)	*E. kobei*	ND	C (1)	AmpC (1)
uni (*n* = 1)	*E. hormaechei* spp. *steigerwaltii*	ND	F (1)	AmpC (1)
uni (*n* = 1)	*E. hormaechei* spp. *steigerwaltii*	ND	E (1)	*bla*_CTX-M-15_ (1)

Abbreviations: *n*—number of isolates; A—University hospital Plovdiv; B—University hospital Varna; C—University hospital Pleven; D—First Sofia hospital; E—Second Sofia hospital; F—Third Sofia hospital; uni—unique ERIC profile; AmpC—AmpC hyperproducer.

## Supplementary Materials

The following supporting information can be downloaded at: https://www.mdpi.com/article/10.3390/microorganisms10112144/s1,: Table S1: Antimicrobial susceptibility of 205 cefotaxime resistant *Enterobacterales* isolates from fecal carriage samples. Table S2: Isoelectric focusing of representative fecal carriage isolates. Figure S1: Isoelectric focusing and bioassay of a *K. pneumoniae* isolate (mixed sequence). Figure S2: ERIC typing of *K. pneumoniae* isolates. Figure S3: ERIC typing of *E. coli* isolates.

## References

[1] Bush K., Bradford P. (2020). Epidemiology of β-Lactamase-producing pathogens. Clin. Microbiol. Rev..

[2] Nordmann P., Poirel L. (2019). Epidemiology and diagnostics of carbapenem resistance in Gram-negative bacteria. Clin. Infect. Dis..

[3] Wyres K.L., Lam M.M.C., Holt K.E. (2020). Population genomics of *Klebsiella pneumoniae*. Nat. Rev. Microbiol..

[4] Adler A., Katz D.E., Marchaim D. (2016). The Continuing Plague of Extended-spectrum β-lactamase-producing Enterobacteriaceae Infections. Infect. Dis. Clin. N. Am..

[5] Rodríguez-Baño J., Gutiérrez-Gutiérrez B., Machuca I., Pascual A. (2018). Treatment of Infections Caused by Extended-Spectrum-Beta-Lactamase-, AmpC-, and Carbapenemase-Producing Enterobacteriaceae. Clin. Microbiol. Rev..

[6] Rahman S.U., Ali T., Ali I., Khan N.A., Han B., Gao J. (2018). The Growing Genetic and Functional Diversity of Extended Spectrum Beta-Lactamases. BioMed Res. Int..

[7] Woodford N., Turton J.F., Livermore D.M. (2011). Multiresistant Gram-negative bacteria: The role of high-risk clones in the dissemination of antibiotic resistance. FEMS Microbiol. Rev..

[8] Partridge S.R., Kwong S.M., Firth N., Jensen S.O. (2018). Mobile Genetic Elements Associated with Antimicrobial Resistance. Clin. Microbiol. Rev..

[9] Woerther P.L., Burdet C., Chachaty E., Andremont A. (2013). Trends in human fecal carriage of extended-spectrum β-lactamases in the community: Toward the globalization of CTX-M. Clin. Microbiol. Rev..

[10] Karanika S., Karantanos T., Arvanitis M., Grigoras C., Mylonakis E. (2016). Fecal Colonization with Extended-spectrum Beta-lactamase-Producing *Enterobacteriaceae* and Risk Factors Among Healthy Individuals: A Systematic Review and Metaanalysis. Clin. Infect. Dis..

[11] Jolivet S., Vaillant L., Poncin T., Lolom I., Gaudonnet Y., Rondinaud E., Bendjelloul G., Lomont A., Lucet J.C., Armand-Lefèvre L. (2018). Prevalence of carriage of extended-spectrum β-lactamase-producing enterobacteria and associated factors in a French hospital. Clin. Microbiol. Infect..

[12] Hoffmann H., Roggenkamp A. (2003). Population genetics of the nomenspecies *Enterobacter* cloacae. Appl. Environ. Microbiol..

[13] Nordmann P., Jayol A., Poirel L. (2016). Universal Culture Medium for Screening Polymyxin-Resistant Gram-Negative Isolates. J. Clin. Microbiol..

[14] Jarlier V., Nicolas M.H., Fournier G., Philippon A. (1988). Extended broad-spectrum beta-lactamases conferring transferable resistance to newer beta-lactam agents in *Enterobacteriaceae*: Hospital prevalence and susceptibility patterns. Rev. Infect. Dis..

[15] Matthew M., Harris A.M., Marshall M.J., Ross G.W. (1975). The use of analytical isoelectric focussing for detection and identification of β-lactamases. J. Gen. Microbiol..

[16] Markovska R., Stoeva T., Schneider I., Boyanova L., Popova V., Dacheva D., Kaneva R., Bauernfeind A., Mitev V., Mitov I. (2015). Clonal dissemination of multilocus sequence type ST15 KPC-2-producing *Klebsiella pneumoniae* in Bulgaria. APMIS.

[17] Poirel L., Walsh T.R., Cuvillier V., Nordmann P. (2011). Multiplex PCR for detection of acquired carbapenemase genes. Diagn. Microbiol. Infect. Dis..

[18] Rasheed J.K., Kitchel B., Zhu W., Anderson K.F., Clark N.C., Ferraro M.J., Savard P., Humphries R.M., Kallen A.J., Limbago B.M. (2013). New Delhi metallo-β-lactamase-producing *Enterobacteriaceae,* United States. Emerg. Infect. Dis..

[19] Pérez-Pérez F.J., Hanson N.D. (2002). Detection of plasmid-mediated AmpC beta-lactamase genes in clinical isolates by using multiplex PCR. J. Clin. Microbiol..

[20] Versalovic J., Koeuth T., Lupski J. (1991). Distribution of repetitive DNA sequences in eubacteria and application to fingerprinting of bacterial genomes. Nucleic Acids Res..

[21] Markovska R., Stoeva T., Boyanova L., Stankova P., Schneider I., Keuleyan E., Mihova K., Murdjeva M., Sredkova M., Lesseva M. (2019). Multicentre investigation of carbapenemase-producing *Klebsiella pneumoniae* and *Escherichia coli* in Bulgarian hospitals—Interregional spread of ST11 NDM-1-producing *K. pneumoniae*. Infect. Genet. Evol..

[22] Clermont O., Dhanji H., Upton M., Gibreel T., Fox A., Boyd D., Mulvey M.R., Nordmann P., Ruppé E., Sarthou J.L. (2009). Rapid detection of the O25b-ST131 clone of *Escherichia coli* encompassing the CTX-M-15-producing strains. J. Antimicrob. Chemother..

[23] Clermont O., Bonacorsi S., Bingen E. (2000). Rapid and simple detection of the *Escherichia coli* phylogenetic group. Appl. Environ. Microbiol..

[24] Magiorakos A.P., Srinivasan A., Carey R.B., Carmeli Y., Falagas M.E., Giske C.G., Harbarth S., Hindler J.F., Kahlmeter G., Olsson-Liljequist B. (2012). Multidrug-resistant, extensively drug-resistant and pandrug-resistant bacteria: An international expert proposal for interim standard definitions for acquired resistance. Clin. Microbiol. Infect..

[25] Markovska R., Stankova P., Stoeva T., Ivanova D., Pencheva D., Kaneva R., Boyanova L. (2021). Fecal Carriage and Epidemiology of Extended-Spectrum Beta-Lactamase/Carbapenemases Producing *Enterobacterales* Isolates in Bulgarian Hospitals. Antibiotics.

[26] Aires-de-Sousa M., Lopes E., Gonçalves M.L., Pereira A.L., Machado E., Costa A., de Lencastre H., Poirel L. (2020). Intestinal carriage of extended-spectrum beta-lactamase-producing *Enterobacteriaceae* at admission in a Portuguese hospital. Eur. J. Clin. Microbiol. Infect. Dis..

[27] Pilmis B., Cattoir V., Lecointe D., Limelette A., Grall I., Mizrahi A., Marcade G., Poilane I., Guillard T., Bourgeois N.N. (2018). Carriage of ESBL-producing *Enterobacteriaceae* in French hospitals: The PORTABLSE study. J. Hosp. Infect..

[28] Abdallah H.M., Alnaiemi N., Reuland E.A., Wintermans B.B., Koek A., Abdelwahab A.M., Samy A., Abdelsalam K.W., Vandenbroucke-Grauls C.M. (2017). Fecal carriage of extended-spectrum β-lactamase- and carbapenemase-producing *Enterobacteriaceae* in Egyptian patients with community-onset gastrointestinal complaints: A hospital -based cross-sectional study. Antimicrob. Resist. Infect. Control.

[29] Dimitrova D., Markovska R., Stoeva T., Stankova P., Boyanova L., Mihova K., Kaneva R., Mitov I. (2019). First report of DHA-1 producing *Enterobacter cloacae* isolate in Bulgaria. Folia Med..

[30] David S., Reuter S., Harris S.R., Glasner C., Feltwell T., Argimon S., Abudahab K., Goater R., Giani T., Errico G. (2019). Epidemic of carbapenem-resistant *Klebsiella pneumoniae* in Europe is driven by nosocomial spread. Nat. Microbiol..

[31] De Oliveira Santos J.V., da Costa Júnior S.D., Cavalcanti I.D.L., de Souza J.B., Coriolano D.L., da Silva W.R.C., Alves M.H.M.E., Cavalcanti I.M.F. (2022). Panorama of Bacterial Infections Caused by Epidemic Resistant Strains. Curr. Microbiol..

[32] Yan L., Sun J., Xu X., Huang S. (2020). Epidemiology and risk factors of rectal colonization of carbapenemase-producing Enterobacteriaceae among high-risk patients from ICU and HSCT wards in a university hospital. Antimicrob. Resist. Infect. Control.

[33] Errico G., Gagliotti C., Monaco M., Masiero L., Gaibani P., Ambretti S., Landini M.P., D’Arezzo S., di Caro A., Parisi S.G. (2019). Colonization and infection due to carbapenemase-producing Enterobacteriaceae in liver and lung transplant recipients and donor-derived transmission: A prospective cohort study conducted in Italy. Clin. Microbiol. Infect..

[34] Hameed M.F., Chen Y., Wang Y., Shafiq M., Bilal H., Liu L., Ma J., Gu P., Ge H. (2021). Epidemiological Characterization of Colistin and Carbapenem Resistant Enterobacteriaceae in a Tertiary: A Hospital from Anhui Province. Infect. Drug Resist..

[35] Bilal H., Zhang G., Rehman T., Han J., Khan S., Shafiq M., Yang X., Yan Z., Yang X. (2021). First Report of *bla*_NDM-1_ Bearing IncX3 Plasmid in Clinically Isolated ST11 *Klebsiella pneumoniae* from Pakistan. Microorganisms.

[36] Zimmerman F.S., Assous M.V., Bdolah-Abram T., Lachish T., Yinnon A.M., Wiener-Well Y. (2013). Duration of carriage of carbapenem-resistant *Enterobacteriaceae* following hospital discharge. Am. J. Infect. Control.

[37] Novais Â., Ferraz R.V., Viana M., da Costa P.M., Peixe L. (2022). NDM-1 Introduction in Portugal through a ST11 KL105 *Klebsiella pneumoniae* Widespread in Europe. Antibiotics.

[38] Izdebski R., Sitkiewicz M., Urbanowicz P., Krawczyk M., Gniadkowski M. (2020). Genomic background of the *Klebsiella pneumoniae* NDM-1 outbreak in Poland, 2012–2018. J. Antimicrob. Chemother..

[39] Studentova V., Dobiasova H., Hedlova D., Dolejska M., Papagiannitsis C.C., Hrabak J. (2015). Complete nucleotide sequences of two NDM-1-encoding plasmids from the same sequence type 11 *Klebsiella pneumoniae* strain. Antimicrob. Agents Chemother..

[40] Xu Q., Pan F., Sun Y., Wang C., Shi Y., Zhang T., Yu F., Zhang H. (2020). Fecal Carriage and Molecular Epidemiology of Carbapenem-Resistant *Enterobacteriaceae* from Inpatient Children in a Pediatric Hospital of Shanghai. Infect. Drug Resist..

[41] Hernández-García M., Pérez-Viso B., Carmen Turrientes M., Díaz-Agero C., López-Fresneña N., Bonten M., Malhotra-Kumar S., Ruiz-Garbajosa P., Cantón R. (2018). Characterization of carbapenemase-producing *Enterobacteriaceae* from colonized patients in a university hospital in Madrid, Spain, during the R-GNOSIS project depicts increased clonal diversity over time with maintenance of high-risk clones. J. Antimicrob. Chemother..

[42] Shafiq M., Huang J., Shah J.M., Ali I., Rahman S.U., Wang L. (2021). Characterization and resistant determinants linked to mobile elements of ESBL-producing and mcr-1-positive *Escherichia coli* recovered from the chicken origin. Microb. Pathog..

[43] Rahman S.U., Muhammad N., Ali T., Saddique U., Ahmad S., Shafiq M., Han B. (2021). Genotypic characterization of multidrug resistant *Escherichia coli* isolates reveals co-existence of ESBL- and carbapenemase- encoding genes linked to ISCR1. Vet. Ital..

[44] Rodrigues C., Machado E., Ramos H., Peixe L., Novais Â. (2014). Expansion of ESBL-producing *Klebsiella pneumoniae* in hospitalized patients: A successful story of international clones (ST15, ST147, ST336) and epidemic plasmids (IncR, IncFIIK). Int. J. Med. Microbiol..

[45] Markovska R., Stoeva T., Boyanova L., Stankova P., Pencheva D., Keuleyan E., Murjeva M., Sredkova M., Ivanova D., Lazarova G. (2017). Dissemination of successful international clone ST15 and clonal complex 17 among Bulgarian CTX-M-15 producing *K. pneumoniae* isolates. Diagn. Microbiol. Infect. Dis..

[46] Zhang R., Liu L., Zhou H., Chan E.W., Li J., Fang Y., Li Y., Liao K., Chen S. (2017). Nationwide Surveillance of Clinical Carbapenem-resistant *Enterobacteriaceae* (CRE) Strains in China. EBioMedicine.

[47] Rojas L.J., Weinstock G.M., de La Cadena E., Diaz L., Rios R., Hanson B.M., Brown J.S., Vats P., Phillips D.S., Nguyen H. (2017). An analysis of the epidemic of *Klebsiella* pneumoniae carbapenemase-producing *K. pneumoniae*: Convergence of two evolutionary mechanisms creates the “Perfect Storm”. J. Infect. Dis..

[48] Arzilli G., Scardina G., Casigliani V., Petri D., Porretta A., Moi M., Lucenteforte E., Rello J., Lopalco P., Baggiani A. (2022). Screening for antimicrobial-resistant Gram-negative bacteria in hospitalised patients, and risk of progression from colonisation to infection: Systematic review. J. Infect..

[49] Rogers B.A., Sidjabat H.E., Paterson D.L. (2011). *Escherichia coli* O25b-ST131: A pandemic, multiresistant, community-associated strain. J. Antimicrob. Chemother..

[50] Can F., Azap O.K., Seref C., Ispir P., Arslan H., Ergonul O. (2015). Emerging *Escherichia coli* O25b/ST131 clone predicts treatment failure in urinary tract infections. Clin. Infect. Dis..

[51] Mathers A.J., Peirano G., Pitout J.D. (2015). The role of epidemic resistance plasmids and international high-risk clones in the spread of multidrug-resistant *Enterobacteriaceae*. Clin. Microbiol. Rev..

[52] Overdevest I., Haverkate M., Veenemans J., Hendriks Y., Verhulst C., Mulders A., Couprie W., Bootsma M., Johnson J., Kluytmans J. (2016). Prolonged colonisation with *Escherichia coli* O25:ST131 versus other extended-spectrum beta-lactamase-producing *E. coli* in a long-term care facility with high endemic level of rectal colonisation, the Netherlands, 2013 to 2014. Eurosurveillance.

[53] López-Cerero L., Salamanca E., Delgado-Valverde M., Rodríguez-Martínez J.M., Rodríguez-Baño J., Pascual Á. (2022). Higher prevalence of CTX-M-27-producing *Escherichia coli* belonging to ST131 clade C1 among residents of two long-term care facilities in Southern Spain. Eur. J. Clin. Microbiol. Infect. Dis..

[54] Adler A., Gniadkowski M., Baraniak A., Izdebski R., Fiett J., Hryniewicz W., Malhotra-Kumar S., Goossens H., Lammens C., Lerman Y. (2012). Transmission dynamics of ESBL-producing *Escherichia coli* clones in rehabilitation wards at a tertiary care centre. Clin. Microbiol. Infect..

[55] Fernández-Martínez M., González-Rico C., Gozalo-Margüello M., Marco F., Gracia-Ahufinger I., Aranzamendi M., Sánchez-Díaz A.M., Vicente-Rangel T., Chaves F., Calvo Montes J. (2021). Molecular characterization of multidrug resistant Enterobacterales strains isolated from liver and kidney transplant recipients in Spain. Sci. Rep..

[56] Fox S., Goswami C., Holden M., Connolly J.P.R., Mordue J., O’Boyle N., Roe A., Connor M., Leanord A., Evans T.J. (2020). A highly conserved complete accessory *Escherichia coli* type III secretion system 2 is widespread in bloodstream isolates of the ST69 lineage. Sci. Rep..

[57] Poirel L., Savov E., Nazli A., Trifonova A., Todorova I., Gergova I., Nordmann P. (2014). Outbreak caused by NDM-1- and RmtB-producing *Escherichia coli* in Bulgaria. Antimicrob. Agents Chemother..

[58] Chowdhury P.R., McKinnon J., Liu M., Djordjevic S.P. (2019). Multidrug Resistant uropathogenic *Escherichia coli* ST405 with a novel, composite IS*26* transposon in a unique chromosomal location. Front. Microbiol..

[59] Peirano G., Matsumura Y., Adams M.D., Bradford P., Motyl M., Chen L., Kreiswirth B.N., Pitout J.D.D. (2018). Genomic Epidemiology of global carbapenemase-producing *Enterobacter* spp., 2008–2014. Emerg. Infect. Dis..

[60] Izdebski R., Baraniak A., Zabicka D., Sekowska A., Gospodarek-Komkowska E., Hryniewicz W., Gniadkowski M. (2018). VIM/IMP carbapenemase-producing Enterobacteriaceae in Poland: Epidemic *Enterobacter hormaechei* and *Klebsiella oxytoca* lineages. J. Antimicrob. Chemother..

