# Supplementary material for: Dissemination of High-Risk Clones Enterobacterales among Bulgarian Fecal Carriage Isolates
Source: Microorganisms. 2022 Oct 29;10(11):2144. doi: 10.3390/microorganisms10112144 (PMC9696877; doi:10.3390/microorganisms10112144)
Supplement: Supplementary file 1 [file microorganisms-10-02144-s001.zip › microorganisms-1961655-supplementary.pdf]

Table S1. Antimicrobial susceptibility of 205 cefotaxime resistant *Enterobacterales* isolates from fecal carriage samples.

**Antimicrobial agent**	**Tested isolates**	**S**	**R+I**
Amoxicillin/clavulanic acid	205	6 (7%)	191 (93%)
Cefoxitine	205	133 (65%)	72 (35%)
Cefotaxime	205	0	100 (100%)
Ceftazidime	205	28(14%)	177 (86%)
Cefepime	205	5 (5%)	200 (95%)
Imipenem	205	191 (93%)	14 (7%)
Meropenem	205	191 (93%)	14 (7%)
Gentamicin	205	90 (44%)	115 (56%)
Amikacin	205	103 (50%)	102 (50%)
Tobramycin	205	66 (32%)	139 (68%)
Ciprofloxacin	205	5 (5%)	95 (95%)
Levofloxacin	205	68(33%)	137 (67%)
Trimethoprim/sulfamethoxazole	100	99 (48%)	106 (52%)
Chloramphenicol*	100	159 (78%)	46 (22%)
Tigecycline	103*	86 (83%)	17 (17%)
Tigecycline	73**	5 (7%)	68 (93%)
Fosfomycin	103*	100 (97%)	3 (3%)
Colistin	205	200 (97,6%)	5 (2,4%)

Abbreviations: *, susceptibility to tigecycline and fosfomycin for 103 *E. coli* isolates was determined with disks-diffusion method; **, susceptibility to tigecycline for 73 *K. pneumoniae* isolates was determined by MIC strip test

Table S2. Isoelectric focusing of representative fecal carriage isolates

β-lactamase	Positive isolates according species (number)	Number isolates, tested with IEF	pI of the beta-lactamases with CTX hydrolytic activity
bla$_{CTX-M-15}$ *n*=84	*Klebsiella* spp. (24)<br>*E. coli* (46)<br>*Enterobacter* spp. (7)<br>*C. freundii* (6)<br>*M. morganii (*1)	*n*=4<br>*n*=9<br>*n*=2<br>*n*=1<br>*n*=1	8.8
bla$_{CTX-M-3}$ *n*=49	*Klebsiella* spp. (30)<br>*E. coli* (18)<br>*Enterobacter* sp. (1)	*n*=12<br>*n*=4<br>*n*=1	8.4
bla$_{CTX-M-9}$ *n*=1	*E. coli* (1)	*n*=1	8.1
bla$_{CTX-M-14}$ *n*=8	*E. coli* (7)<br>*Klebsiella* sp. (1)	*n*=1<br>*n*=1	8.1
bla$_{CTX-M-27}$ *n*=22	*E. coli* (22)	*n*=8	8.2
bla$_{NDM-1}$ + *bla*$_{CTX-M-15}$ + *bla*$_{CMY-4}$ *n*=9	*Klebsiella* spp. (9)	*n*=3	8.8 and 9.2
bla$_{NDM-1}$ + *bla*$_{CTX-M-3}$ + *bla*$_{CMY-4}$ *n*=1	*Klebsiella* sp. (1)	*n*=1	8.4 and 9.2
bla$_{KPC-2}$ *n*=1	*Klebsiella* sp. (1)	*n*=1	6.7
bla$_{KPC-2}$ + *bla*$_{CTX-M-15}$ *n*=1	*Klebsiella* sp. (1)	*n*=1	6.7^{χ} and 8.8
bla$_{KPC-2}$ + *bla*$_{CTX-M-3}$ *n*=1	*Klebsiella* sp. (1)	*n*=1	6.7^{χ} and 8.4
bla$_{DHA-1}$ *n*=5	*E. coli* (5)	*n*=3	7.8*
bla$_{CMY-2}$ *n*=2	*E. coli* (2)	*n*=2	> 8.8
hyperproduction of SHV-1 *n*=1	*Klebsiella* sp. (1)	*n*=1	7.6*
Unknown mechanism *n*=3	*E. coli* (1)<br>*Klebsiella* spp. (2)	*n*=1<br>*n*=1	9.2<br>8.0*
Isolate with mixed sequence	*Klebsiella* sp. (1)	*n*=1	8.4 and 8.8

Abreviations: * didn't show cefotaxime hydrolyzing activity, χ have in addition imipenem hydrolytic activity

Figure S1 A. Isoelectric focusing and bioassay of a *K. pneumoniae* isolate (mixed sequence)

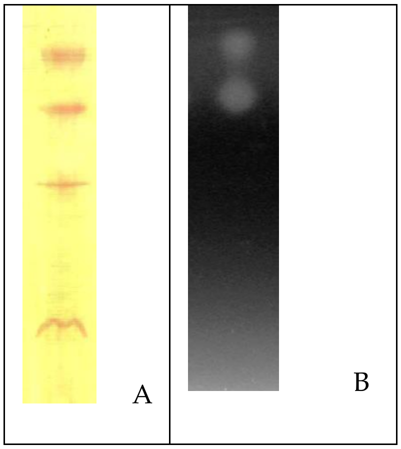

Abbreviations: A. Isoelectric focusing of a *K. pneumoniae* isolate – four beta-lactamases were visible with nitrocefin staining – with pI 5.4, with pI 7.6, pI 8.4 and pI 8.8 (pI were detected with comparison with control isolates with known enzymes); B. Bioassay - Bands with pI 8.8 and pI 8.4 had CTX hydrolytic activity

Figure S2. ERIC typing of *K. pneumoniae* isolates

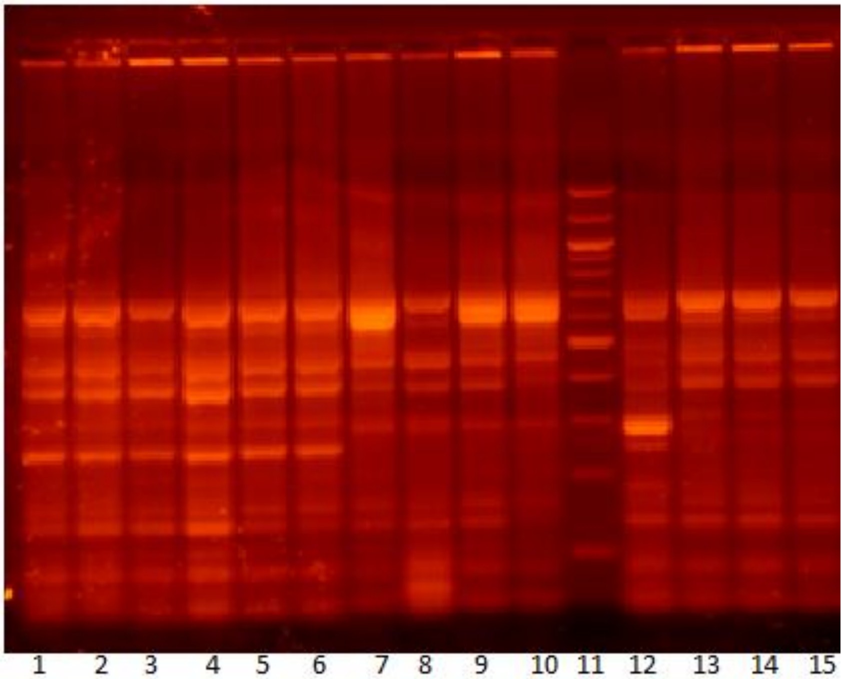

Abbreviations: Clone p - lines 1 - 6 ; clone a – lines 7, 9, 10 clone b – lines 13-16, clone h – line 8, clone c – line 12, DNA ladder - line 11

Figure S3. ERIC typing of *E. coli* isolates

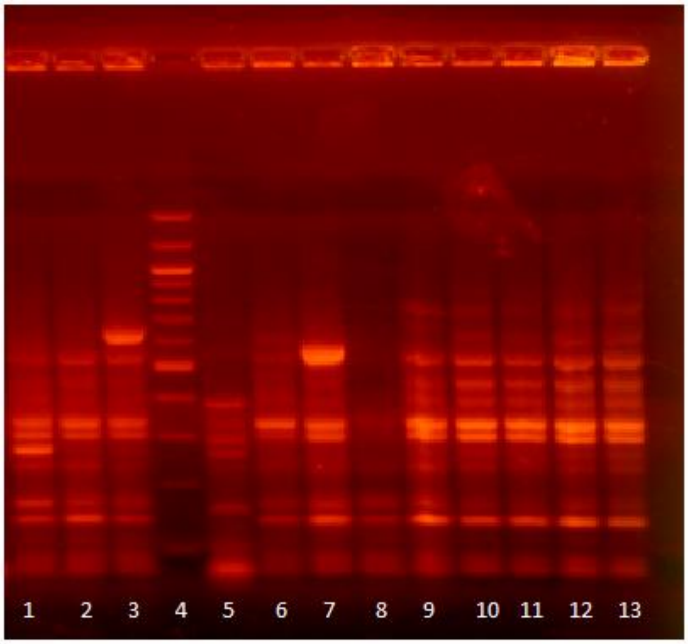

Abbreviations: clone B – line 1, Clone A – lines 2, 9 -13, clone A1– line 3; clone S – line 5, clone A2 – line 6; clone A 3 – line 7; clone A5 – line 8; line 4 – DNA ladder
